# Evaluation of Nutrition Knowledge in Female Gaelic Games Players

**DOI:** 10.3390/sports8120154

**Published:** 2020-11-29

**Authors:** Michèle Renard, David T. Kelly, Niamh Ní Chéilleachair, Ciarán Ó Catháin

**Affiliations:** Department of Sport and Health Sciences, Athlone Institute of Technology, N37 HD68 Athlone, Ireland; davidkelly@ait.ie (D.T.K.); nnicheilleachair@ait.ie (N.N.C.); ciaranocathain@ait.ie (C.Ó.C.)

**Keywords:** questionnaire, survey, education, dietary knowledge, team sport, sports nutrition

## Abstract

Improvements in nutrition knowledge have been associated with increased carbohydrate consumption and greater adherence to dietary recommendations among female athletes. In order to assess whether nutrition knowledge interventions in female Gaelic games players may be beneficial, it is necessary to first of all investigate current levels of nutrition knowledge in this population. Given that many demographic characteristics have been shown to influence nutrition knowledge, it is also important for these to be investigated. The aims of this study were to evaluate the nutrition knowledge of female Gaelic games players, compare knowledge by players’ characteristics and identify players’ preferences for information and support. A validated 35-item questionnaire was completed by 328 female Gaelic games players (Age: 23.7 ± 5.0 years). Players’ mean nutrition knowledge score was 46.0% ± 11.8% and classified as “poor”. Elite players scored greater (+4.5–5.9%, *p* < 0.05) than subelite players. Players with higher levels of general education, history of formal nutrition education and previous advice from a nutritionist also presented greater nutrition knowledge (+3.7–7.5%, *p* < 0.05). Future education interventions with female Gaelic games players may lead to beneficial changes in dietary behaviour and would likely benefit from stratifying content based on athletes’ demographic characteristics, given the differences observed.

## 1. Introduction

Gaelic games are a group of sports indigenous to Ireland that include Gaelic football, Camogie, hurling, court handball, road bowls and various individual athletic events [1]. Gaelic football is the most popular of the Gaelic games, played by both males and females [1,2], whilst camogie is the female equivalent of hurling [3]. Both sports are contested by two teams of fifteen players and are classified as high-intensity intermittent invasion field games with match-play, including frequent bouts of high-speed running, tackling and jumping with brief recovery intervals [1]. What separates the sports from each other is the use of a ball similar to soccer in Gaelic football [4], and the use of a stick known as a hurley and a ball called a sliotar in Camogie [5]. With the increasing popularity of both sports, scientific publications with regards to female players physiological profiles [2,6] as well as injury incidence and prevention [3,5,7] have been produced. However, nutritional investigations are currently lacking, and such information may prove useful to inform future interventions designed to improve dietary intake.

Adequate dietary intake is essential to optimise performance during game play and support physiological adaptation from training [8]. Despite this, female field-based team sport players’ diets have been shown to be insufficient in energy intake surrounding both competition [9,10] and training, whilst consistently failing to meet recommendations for carbohydrate intake [9,10,11,12,13,14,15]. Numerous factors can influence the dietary intake of athletes, including taste and food preference, cultural beliefs and nutrition knowledge [16]. Significant positive associations (*r* = 0.05–0.261) have been identified between an athlete’s nutrition knowledge and dietary intake [17,18,19]. Although these associations are weak, increases in knowledge have been associated with improvements in dietary intake previously [20,21,22]. A dietician-led nutrition education intervention amongst a cohort of collegiate volleyball players led to improvements of 12.4% in nutrition knowledge score, 24% in total energy intake, 36% in carbohydrate intake and 22% in protein intake [22]. Collegiate swimmers and soccer players displayed increases in nutrition knowledge with reported improvements in dietary intake [21], and a peer-based nutrition education programme amongst collegiate female athletes led to an average 7.7% increase in nutrition knowledge score [20]. It must be acknowledged that, in the aforementioned investigations [20,21,22] and the wider literature, the direct influence of increases in nutrition knowledge on performance has not been investigated. Despite this, the dietary intake of male Gaelic football players [23] and female Australian rules players [13,14] has demonstrated inadequate carbohydrate intake to support optimal performance and recovery. Investigations in other team sports athletes have identified improvements in performance when carbohydrate intake was increased [24,25]. Therefore, improvements in nutrition knowledge could serve as a mechanism for dietary behaviour change, which, in turn, may elicit improvements in performance.

Previous research has identified that female Gaelic football and Camogie players demonstrated inadequate nutrition knowledge (mean score = 55.2%); however, findings are limited to a single study with a low sample size (*n* = 44) and did not assess important demographic characteristics related to nutrition knowledge [26]. General education level, previous nutrition education and age have all previously been associated with higher nutrition knowledge scores [27,28,29,30,31,32]. In contrast, conflicting reports exist regarding the influence of competitive level, type of sport and gender [17,29,32,33], suggesting that their impact on nutrition knowledge may be largely scenario-dependent. The assessment of demographic factors that may influence nutrition knowledge is therefore warranted among female Gaelic games players. It is also important to identify players’ preference for sources of nutrition information and nutritional support services, as this may likely influence their current nutrition knowledge and receptiveness to future advice [34].

With the above factors considered, this study’s aims were to (i) evaluate the nutrition knowledge of female Gaelic games players and identify any areas for improvement, (ii) evaluate the relationship between nutrition knowledge, and the players’ demographic characteristics, and (iii) identify players’ preferences for nutrition information and support.

## 2. Materials and Methods

### 2.1. Partcipants

Female Gaelic games players (*n* = 328, 23.7 ± 3.0 years, 65.8 ± 9.1 kg, 1.66 ± 0.08 m), participating in Gaelic football (*n* = 214) and Camogie (*n* = 114), competing at both club level (subelite, *n* = 218) and intercounty level (elite, *n* = 110) were recruited. Recruitment and data collection were conducted between December 2019 and May 2020. All participants provided informed consent, ethical approval was obtained from the review board (Approval code: 0191102) at the Athlone Institute of Technology (Ireland), and all procedures were completed in accordance with the declaration of Helsinki.

### 2.2. Study Design

The nutrition knowledge of participants was assessed using the validated abridged nutrition for sports knowledge questionnaire (A-NSKQ) [35,36]. The abridged questionnaire was used to facilitate shorter completion times, and thus higher completion rates [35,37]. The questionnaire consists of 35 questions (TOTAL), 11 of which focus on the assessment of general nutrition knowledge (GNK) and the remaining 24 assess sports nutrition knowledge (SNK). Scores are presented as a percentage for TOTAL and each subsection (GNK & SNK) and classified by the following: “poor” (0–49%), “average” (50–65%), “good” (66–75%) and “excellent” knowledge (76–100%) [38]. The questionnaire was delivered online, to enhance participant experience and facilitate greater access in comparison to paper-based methods [39]. A URL link for the questionnaire was shared online via social media outlets (Facebook, Twitter) and Gaelic Athletic Association (GAA) club websites. This link was accessible for a duration of 6 months (December 2019–May 2020). In addition to the A-NSKQ, questions were asked to capture demographic and information source data, as previously described [34,35]. These included: age, stature, mass, highest level of education, previous history of nutrition education, current levels of nutrition support and preferences for nutrition information.

### 2.3. Statistical Analysis

Normality for each category of nutrition knowledge scores (TOTAL, GNK and SNK) was assessed using Shapiro–Wilk’s test, with homogeneity of variances assessed using Levene’s test. A two-way ANOVA was performed to examine the effects of sport (Gaelic football = 214 and Camogie = 114) and playing level (subelite = 218 (Gaelic football = 144, Camogie = 74), and elite = 110 (Gaelic football = 70, Camogie = 40)) on nutrition knowledge scores. In the absence of an interaction effect, the main effects were analysed and reported. All following analyses were performed with Gaelic football and Camogie players as an aggregate sample due to the homogeneity in nutrition knowledge scores observed. Difference in nutrition knowledge scores between formal nutrition education (with = 89, without = 239), any history of previous advice from a nutritionist (yes = 140, no = 188), and a specific preference for private nutritional consultations from a nutritionist instead of other support such as group presentations or cooking classes (yes = 94, no = 234), were analysed using independent sample T-tests. Effect size was reported as Cohen’s d, and interpreted as small (d = 0.2), medium (d = 0.5) and large (d = 0.8) [40]. Differences in scores between age (18–24 = 215, 25–30 = 83, 31+ = 30), highest level of education (high school/second level = 132, undergraduate/third level = 138, post-graduate = 58), and access to nutrition support (information only = 66, information and nutritionist = 53, neither = 209) were analysed using one-way ANOVAs, with Tukey’s post hoc test; if the homogeneity of variance was violated, the Welch ANOVA was interpreted with Games–Howell post hoc. Effect size was reported as n^2^, and interpreted as small (n^2^ = 0.010), medium (n^2^ = 0.060) and large (n^2^ = 0.140) [40]. When parametric assumptions were violated, Mann–Whitney U tests and Kruskal–Wallis H tests were used, with comparisons of group medians. Effect size for Mann–Whitney U test results were reported as r^2^, and interpreted as small (r^2^ = 0.1–0.3), medium (r^2^ = 0.3–0.5) and large (r^2^ ≥ 0.5) [40]. Post hoc for Kruskal–Wallis H test was performed using multiple Mann–Whitney U tests between groups. Data were reported as mean and standard deviations. Statistical analysis was performed using IBM SPSS statistical software for Mac Version 24.0 (IBM corporation, Armonk, NY, USA), with alpha set at *p* < 0.05 for all tests.

## 3. Results

### 3.1. Participants

The online questionnaire link was accessed by a total of 1591 individuals; however, only 328 provided a complete and valid response. Therefore, the invalid responses (1263) were disregarded, and the total sample consisted of 328 female Gaelic games players. Of note, 65.2% (214) of respondents recorded Gaelic football as their primary sport with the remaining 34.8% (114) recording Camogie, and 66.5% (218) of the sample compete at club level (subelite) and 33.5% (110) at county level (elite). Full demographic subgroups are outlined in Table 1.

### 3.2. Overall Nutrition Knowledge

The mean total nutrition knowledge score of female Gaelic games players (*n* = 328) was 46.0% ± 11.8%, classified as “poor”. Scores for each subgroup are presented in Table 1.

### 3.3. General Nutrition Knowledge

Mean score for general nutrition knowledge (GNK) was 58.2 ± 15.6%, classified as “average”. A majority of participants correctly identified whether foods such as cheddar cheese (91.5%), margarine (87.8%) and honey (70.1%) were high or low in fat. Almost all were aware that alcohol consumption can lead to weight gain (98.2%). Less than half of participants knew that “the body has a limited ability to use protein for muscle protein synthesis” (41.2%) and that “eggs contain all the essential amino acids needed by the body” (41.2%). Only 13.1% correctly answered that “Thiamine (Vitamin B1) is needed to take oxygen to muscles”, as false.

### 3.4. Sports Nutrition Knowledge

Mean score for sports nutrition knowledge (SNK) was 40.4 ± 13%, classified as “poor”. Note that 68% percent of participants correctly answered that 1 medium banana contains insufficient carbohydrate for recovery from exercise, 68.9% correctly identified that 100 g of chicken breast contains enough protein to promote muscle growth after resistance exercise, and 67.4% correctly agreed that vegetarian athletes can meet protein requirements without supplements. Also, 32% were aware of carbohydrate requirements for exercise lasting 60–90 min, and 34.5% knew the recommendation for protein intake after a bout of resistance exercise. Few participants answered questions with regards to vitamin and mineral requirements for athletes correctly. For example, only 14.9% and 18.0% correctly disagreed to the statements “the optimal calcium intake for athletes aged 15–24 years is 500 mg” and “Vitamin C should always be taken by athletes”, respectively. Knowledge of hydration was limited, with only 8.8% correctly identifying that athletes should drink water to maintain blood plasma volume.

### 3.5. Type of Sport and Competitive Level

**Figure 1:**
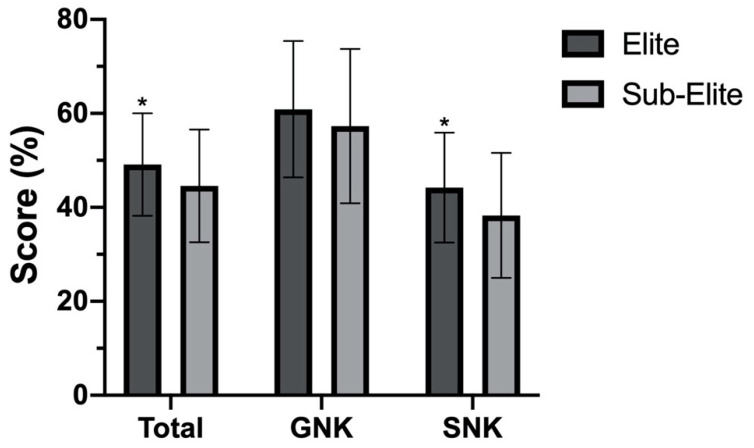
Total, GNK and SNK score compared by competitive playing level. Data are mean ± SD, Total = overall A-NSKQ score, GNK = general nutrition knowledge subscore, SNK = sports nutrition knowledge subscore, * (*p* < 0.05) between elite and subelite for Total and SNK.

There was no statistically significant interaction between sport and competitive level for Total (F (1, 324) = 0.081, *p* = 0.776), GNK (F (1, 324) = 0.085, *p* = 0.771) or SNK (F (1, 324) = 0.046, *p* = 0.830). There were no significant differences between Gaelic football and Camogie players for Total (F (1, 324) = 0.000, *p* = 0.991), GNK (F (1, 324) = 0.198, *p* = 0.657) or SNK (F (1, 324) = 0.054, *p* = 0.816). There was a statistically significant main effect for competitive level, with elite-level players scoring higher (49.1%, 17.2 ± 3.8) than subelite players (44.6%, 15.6 ± 4.2), (F (1, 324) = 10.421, *p* = 0.001, partial n^2^ = 0.031) for Total Score and scoring higher (44.2%, 10.6 ± 2.8) than subelite players (38.3%, 9.2 ± 3.2), (F (1, 324) = 12.278, *p* = 0.001, partial n^2^ = 0.037) for SNK subtotal score, presented in Figure 1.

### 3.6. Age and Education Level

**Figure 2:**
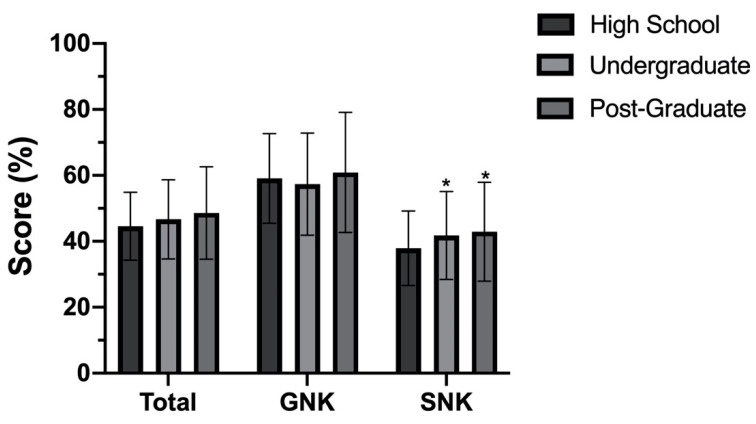
Total, GNK and SNK score compared by education level. Data are mean ± SD, Total = overall A-NSKQ score, GNK = general nutrition knowledge subscore, SNK = sports nutrition knowledge subscore, * (*p* < 0.05) between high school and undergraduate and high school and post-graduate for SNK.

There were no significant differences between age groups for Total (F (2, 325) = 0.708, *p* = 0.493), GNK (X^2^ (3) = 3.028, *p* = 0.220) or SNK (X^2^ (3) = 1.181, *p* = 0.554) scores. There was a significant difference between education level groups for SNK score (X^2^ (3) = 10.679, *p* = 0.005, n^2^ = 0.027). Post hoc analysis revealed significant differences between high school/second level (median score: 9.00) and undergraduate/third level (median score: 10.00) (*p* = 0.004, r^2^ = 0.031) and high school/second level and post-graduate (median score: 10.00) (*p* = 0.013, r^2^ = 0.033). These results are presented in Figure 2 below.

### 3.7. Nutrition Education

Those with formal nutrition education scored higher than those without, for Total (median score: with = 18.00, without = 16.00, *U* = 6900.0, *z* = −4.905, *p* = 0.001, r^2^ = 0.073), GNK (median score: with = 7.00, without = 6.00, *U* = 8709.0, *z* = −2.567, *p* = 0.010, r^2^ = 0.020) and SNK scores (median score: with = 11.00, without = 9.00, *U* = 6947.0, *z* = −4.854, *p* = 0.001, r^2^ = 0.072).

### 3.8. Support Access

Full characteristics and scores based on access to support, previous advice and preferred information source can be found in Table 2. Of the participants, 20.1% reported having access to nutrition information only, 16.2% had access to nutrition information and a nutritionist, and the remaining 63.7% indicated no access to either. There were no significant differences between access to support groups for Total (F (2, 325) = 2.261, *p* = 0.106), GNK (X^2^ (3) = 1.143, *p* = 0.565) or SNK (X^2^ (3) = 3.217, *p* = 0.200) scores.

### 3.9. Previous Advice

The top five most frequently reported sources of previous nutritional advice indicated by participants included: nutritionist (42.7%), family (37.8%), friends (35.1%), teammates (34.8%) and athletic trainer (27.7%). Those that indicated previous advice from a nutritionist scored higher, (48.3%, 16.9 ± 3.7) than those that did not (44.6%, 15.6 ± 4.3), for Total (1.3, 95% CI 0.439 to 2.227, *t* (326) = 2.933, *p* = 0.004, d = 0.327) and SNK (median score: previous advice = 10.00, no previous advice = 9.00, *U* = 10839.0, *z* = −2.746, *p* = 0.006, r^2^ = 0.023) scores. No other comparisons of previous advice source presented a significant difference in Total, GNK or SNK scores.

### 3.10. Information Source Preference

Participants ranked their preferred source of nutritional information on a scale of one to three. The most frequently ranked number one source was a nutritionist (28.0%), followed by an internet search (16.8%) and a dietician (11.9%). Comparisons of preferred information source, ranked number one or not, presented no significant difference in Total, GNK or SNK scores.

### 3.11. Support Wanted

Participants ranked the types of nutritional support they believed to be most useful on a scale of one to five. The most frequently ranked number one (most useful) support was sports nutrition advice (39.9%), followed by private consultations with a nutritionist/dietician (28.7%), healthy eating advice (22.6%), group presentations (5.8%) and cooking classes (3.0%). Those that indicated private consultations with a nutritionist/dietician as the most useful form of support scored higher (49.1%, 17.2 ± 4.0) than those that did not (44.9%, 15.7 ± 4.1), for Total (1.5, 95% CI 0.552 to 2.506, *t* (326) = 3.080, *p* = 0.002, d = 0.376) and SNK (median score: ranked most useful = 11.00, did not = 9.00, *U* = 8488.5, *z* = −3.248, *p* = 0.001, r^2^ = 0.032) scores.

## 4. Discussion

This study aimed to evaluate the nutrition knowledge of female Gaelic games players, comparing scores based on demographic characteristics, while also identifying preferences for nutrition information and support.

Female Gaelic games players (*n* = 328) mean total A-NSKQ score was 46.0% ± 11.8% and classified as “poor”. This was lower than scores previously reported for a smaller cohort of players (*n* = 44, 55.2%) [26] who were assessed using a different tool [41]. When compared to athletic groups assessed using the same tool [35,38], similarities are present with both male (47%, *n* = 177) [35] and female (50.6%, *n* = 26) [32] Australian Rules Football players; however, the latter utilised the unabridged 89-item version of the questionnaire [38]. Further comparison with investigations that used different tools reveals higher nutrition knowledge scores among elite distance runners (64%) [28] and professional rugby players (73%) [42]. A possible explanation for the lower scores among female Gaelic games players may be the fact that they compete on a fully amateur basis [4]. The disparity in available support for players as a result of this may contribute to them scoring poorly in comparison to athletes from other sports competing professionally. Therefore, female Gaelic games players may benefit from the provision of nutrition education resources where viable.

Female Gaelic games players mean GNK score was 58.2% ± 15.6% and classified as “average”. This was also similar to male Australian football players (59%) [35], and provides further evidence to confirm that the previously noted higher nutrition knowledge of females in the general population [17] is unfounded when examining athletic populations [33]. Similar to previous investigations [32], many athletes answered food-based questions correctly, and performed best in subsections related to alcohol. In contrast, participants scored most poorly on questions referring to micronutrients. This again has been identified previously with only 19% of elite and 9% of nonelite Australian athletes correctly selecting “false” to the statement that “Vitamin B-complex helps you to recover faster” [43]. These findings suggest that future nutrition education interventions may benefit from prioritizing topics commonly lacking in knowledge such as micronutrient information.

Female Gaelic games players’ mean SNK score was 40.4 ± 13% and classified as “poor”. This was less than female Australian football players, who scored 51 ± 19% on a sports nutrition subsection [32]; however, they were evaluated using only 8 questions, in comparison to the 24 questions used for female Gaelic games players. Similar to responses for the general nutrition knowledge subsection, participants scored better on food-based questions, yet lacked knowledge surrounding specific macronutrient recommendations. This has been reported previously with many athletes indicating “unsure” when asked to identify protein recommendations in general (53.8%) or specific to post-resistance training intake (42.3%) [32]. If these misconceptions surrounding protein recommendations lead to insufficient protein intake post-exercise, muscle repair and strength/hypertrophy-related adaptions could be diminished [44,45], negatively impacting the performance and recovery of female Gaelic games players. Only 32% of participants were aware of carbohydrate requirements during prolonged exercise (>60–90 min). Similar confusion surrounding carbohydrate recommendations has been found with collegiate athletes from multiple sports (basketball, soccer, track and field) with 53% selecting a value below the current recommendations [46]. These findings further highlight the fact that athletes commonly display good food-based knowledge and may therefore benefit from educational interventions that focus more directly on dietary needs and recommendations for both macro and micronutrients. It may be of particular importance to address the lack of knowledge surrounding carbohydrate requirements, given that carbohydrate is the predominant energy source for the prolonged and intermittent running [47] performed in both Gaelic football and Camogie, and insufficient intake could have profoundly negative consequences for performance [48,49].

The poor nutrition knowledge of female Gaelic games players may have negative consequences for their dietary behaviour. Despite a current lack of dietary investigation within the population, other female field-based team sport athletes’ diets have consistently shown to be insufficient in overall energy and carbohydrate intake [9,10,11,12,13,14,15]. Male Gaelic football players also fail to meet dietary recommendations with average energy deficits of 12.3% per day [50], carbohydrate intakes 1.3–1.6 g/kg per day lower than minimum recommendations [23,50,51] and intakes of fat (31–37.5%) and protein (1.9–2.1 g/kg) towards the higher end of recommendations [23,50,51]. Given the consistency of observations within similar athletic cohorts, the dietary intake of female Gaelic games players is likely to be inadequate for optimal performance and their poor nutrition knowledge could be a significant contributing factor. Future research should assess this hypothesis by performing dietary intake observations in tandem with the assessment of nutrition knowledge.

Investigations that have assessed both nutrition knowledge and dietary intake have revealed poor nutrition knowledge (51–54.5%), and inadequate carbohydrate intakes (2.8–3.2 g/kg) within both male and female Australian rules football players [13,52]. Conversely, rugby players classified with “good” nutrition knowledge were more likely to consume carbohydrate-rich foods and thus meet dietary recommendations in comparison to the players classified with “poor” nutrition knowledge [42]. This highlights the potential positive and negative influences nutrition knowledge can have. Based on this, educational interventions to improve nutrition knowledge scores in female Gaelic games players may result in improved dietary behaviour as previously displayed in volleyball, soccer and baseball players [21,22,53].

Similar to previous findings [33], no difference in nutrition knowledge between sports was identified. Elite (county)-level players scored greater for Total and SNK score in comparison to subelite (club)-level players. This has been identified previously [43], and may be reflective of increased nutritional support/awareness at the higher competitive level; however, no significant differences based on athletes’ access to support were observed and this would require further investigation to confirm. Similar to previous research [27,28,29,30,32], those with higher general education and previous history of nutrition education scored better, therefore future education interventions should consider this.

Players most frequently reported that their source of previous nutritional advice was a nutritionist. Those that indicated previous advice from a nutritionist scored greater for Total and SNK scores in comparison to those that did not. This finding was in contrast to a lack of difference when players were compared by their current access to a nutritionist or not, highlighting both the long-term impact nutritional advice from a certified professional may have and the need to assess the frequency of engagement with nutritional support, in a bid to explain the discrepancy in findings. Female Gaelic games players also indicated their most preferred nutrition information source as a nutritionist (28.0%). However, similar to previous findings [34,54], this did not impact nutrition knowledge scores. As observed in previous research [32,34], participants ranked access to nutrition information specific to their sport, and one-to-one private consultations with a nutritionist/dietician as the types of support they believed to be most useful. Interestingly, those that reported private consultations as the most useful type of support scored greater for Total and SNK in comparison to those that did not. With this in mind, the information and support preferences amongst female Gaelic games players must be considered when designing tailored nutrition education interventions.

The findings presented must be interpreted with the limitations of the study design in mind. Firstly, data collection was restricted to female Gaelic games players within an exclusively Irish context; therefore, the extrapolation of findings to other sports with athletes from different countries may be limited. However, this is acknowledged in light of the studies primary aim to obtain data specific to the target population to inform future nutrition-education interventions. Participants completed the nutrition knowledge assessment online as this has shown to facilitate greater access to participants [39]. Due to this, whether or not participants accessed additional support or resources whilst completing the questionnaire is unclear; however, the average completion time and poor overall scores suggest that this was unlikely. The 35-item A-NSKQ [35,36] was utilised in comparison to the full length 89-item NSKQ [38] due to a 31% greater completion rate in previous research [35]. This likely permitted the recruitment of a larger sample size, yet restricted the extent to which gaps in players nutrition knowledge could be explored in comparison to recent investigations with much smaller sample sizes [32]. Despite variation in scores being observed between groups, all were still classified as either “poor” or “average”. Previous authors [34] have highlighted that this may be due to the questionnaires’ focus on assessing athlete’s declarative knowledge of sports nutrition recommendations, in favor of procedural knowledge, such as recipe planning and meal preparation. This could also explain why athletes who have received professional dietetic support still scored poorly, as the support given may focus on food selection and provision. However, based on the widespread poor quality of dietary intake reported [9,10,11,12,13,14,15], it may be fair to assume that the procedural knowledge of players is also poor, warranting further investigation. The assessment of nutrition knowledge provides important information to support the evaluation of an athlete’s dietary behaviour, particularly given the evidence of its positive influence [21,22,53]. However, as highlighted previously, numerous factors influence dietary intake, and an individual’s knowledge and education alone may not contribute sufficiently to behaviour change [17,55]. Broader investigation of nutrition knowledge and the factors that might influence it, such as dietary preference (omnivorous, vegetarian, vegan) [56], may provide greater insight. Future research should focus upon exploring nutrition knowledge in tandem with dietary intake observation to explore this relationship further, and future nutritional educational interventions would likely benefit from adopting a scientifically supported behavioural change framework [57,58].

## 5. Conclusions

The nutrition knowledge of female Gaelic football and camogie players is poor. Based on previous findings, improving this may yield beneficial changes in dietary behaviour. Focus should be placed on strategies to enhance nutrition knowledge of sport-specific concepts, addressing macro and micronutrient recommendations as players scored poorest on these aspects. Future nutritional education interventions may benefit from stratifying their content based on athlete’s demographic characteristics, given the differences observed.

## Figures and Tables

**Table 1:** Nutrition knowledge scores for total sample and demographic subgroups.

Group	(n)	Total	(%)	GNK	(%)	SNK	(%)
**Total Sample:**	328	16.1 ± 4.1	46.0 ± 11.8	6.4 ± 1.7	58.2 ± 15.6	9.7 ± 3.1	40.4 ± 13.0
**Sport:**							
Football	214	16.1 ± 4.2	46.0 ± 12.0	6.4 ± 1.7	58.2 ± 15.0	9.7 ± 3.3	40.4 ± 13.7
Camogie	114	16.2 ± 4.0	46.3 ± 11.3	6.5 ± 1.8	59.1 ± 16.6	9.7 ± 2.8	40.4 ± 11.6
**Age (yrs):**							
18–24	215	16.0 ± 3.8	45.7 ± 10.9	6.4 ± 1.6	58.2 ± 14.7	9.6 ± 2.9	40.0 ± 12.2
25–30	83	16.3 ± 4.8	46.6 ± 13.8	6.4 ± 1.9	58.2 ± 17.3	9.9 ± 3.6	41.3 ± 15.1
31+	30	16.9 ± 4.2	48.3 ± 11.9	6.8 ± 1.9	61.8 ± 17.2	10.0 ± 3.0	41.7 ± 12.5
**Nutrition Education:**							
Yes	89	17.9 ± 3.8 *	51.1 ± 10.9	6.9 ± 1.7 *	62.7 ± 15.4	11.0 ± 2.8 *	45.8 ± 11.5
No	239	15.5 ± 4.0	44.3 ± 11.6	6.3 ± 1.7	57.3 ± 15.5	9.2 ± 3.1	38.3 ± 12.9

Note: Data are mean ± SD, Total = overall A-NSKQ score, GNK = general nutrition knowledge subscore, SNK = sports nutrition knowledge subscore, * (*p* < 0.05) between nutrition education and no nutrition education for Total, GNK and SNK.

**Table 2:** Nutrition knowledge scores based on previous support and preferred information source.

Group	(n)	Total	(%)	GNK	(%)	SNK	(%)
**Nutrition Support:**							
Information Only	66	16.4 ± 3.8	46.9 ± 10.9	6.6 ± 1.5	60.0 ± 13.2	9.8 ± 3.2	40.8 ± 13.3
Info & Nutritionist	53	17.1 ± 3.5	48.9 ± 10.0	6.7 ± 1.6	60.9 ± 14.4	10.4 ± 2.4	43.3 ± 10.1
None	209	15.8 ± 4.3	45.1 ± 12.4	6.3 ± 1.8	57.3 ± 16.5	9.5 ± 3.2	39.6 ± 13.5
**Previous Advice:**							
Athletic Trainer	91	16.5 ± 4.0	47.1 ± 11.4	6.5 ± 1.8	59.1 ± 16.4	9.9 ± 2.9	41.3 ± 12.2
Nutritionist	140	16.9 ± 3.7 *	48.3 ± 10.6	6.6 ± 1.5	60.0 ± 14.0	10.3 ± 2.9 *	42.9 ± 12.1
Family	124	15.9 ± 4.1	45.4 ± 11.8	6.4 ± 1.8	58.2 ± 16.2	9.5 ± 3.0	39.6 ± 12.5
Friends	115	16.3 ± 4.2	46.6 ± 11.9	6.4 ± 1.7	58.2 ± 15.9	9.9 ± 3.1	41.3 ± 12.9
Teammates	114	16.5 ± 4.1	47.1 ± 11.7	6.6 ± 1.7	60.0 ± 15.1	9.9 ± 3.2	41.3 ± 13.2
**Info Source:**							
Nutritionist	92	16.1 ± 3.9	46.0 ± 11.2	6.4 ± 1.6	58.2 ± 14.5	9.7 ± 3.1	40.4 ± 12.7
Internet Search	55	16.1 ± 4.1	46.0 ± 11.7	6.6 ± 1.8	60.0 ± 16.2	9.5 ± 3.1	39.6 ± 12.8
Dietician	39	16.8 ± 4.6	48.0 ± 13.2	6.4 ± 1.9	58.2 ± 17.0	10.4 ± 3.3	43.3 ± 13.9

Note: Data are mean ± SD, Total = overall A-NSKQ score, GNK = general nutrition knowledge subscore, SNK = sports nutrition knowledge subscore, * (*p* < 0.05) between those that indicated previous advice from a nutritionist and those that did not for Total and SNK.
